# Rosmarinic acid ameliorates HCl-induced cystitis in rats

**DOI:** 10.1371/journal.pone.0288813

**Published:** 2023-07-18

**Authors:** Naoya Kitamura, Yasunori Yamamoto, Naoki Yamamoto, Takatoshi Murase

**Affiliations:** Biological Science Research, Kao Corporation, Tochigi, Japan; Boston University School of Medicine, UNITED STATES

## Abstract

Shiso (*Perilla frutescens var crispa f*. *purprea*) is a traditional medicinal herb that exerts anti-inflammatory effects and alleviates lower urinary tract symptoms. In this study, we examined the effects of rosmarinic acid, a major polyphenol in shiso, on urinary function and the bladder in a rat hydrochloric acid-induced cystitis model. Sprague–Dawley rats were administered intravesically with hydrochloric acid or saline solution (control) to induce cystitis. Afterwards, the rats were administered orally with distilled water or rosmarinic acid for three days and then the intravesical pressure was measured, a stretch stimulation test was performed using the harvested bladder, and histological and biochemical analyses were performed. In addition, we investigated the effects of rosmarinic acid on the expression of inflammation-related molecules in normal human bladder epithelial cells. Rosmarinic acid ameliorated hydrochloric acid-induced shortening of micturition interval by 49%. In hydrochloric acid-treated bladders, significantly more prostaglandin E_2_ was released after stretching; however, rosmarinic acid suppressed its release to control levels. Rosmarinic acid also reduced hydrochloric acid-induced epithelial thickening and the levels of inflammatory molecules in the bladder. Furthermore, rosmarinic acid suppressed interleukin 1β-induced increases in *Cox2* and *Il6* expression in bladder epithelial cells. These findings indicate that rosmarinic acid can ameliorate hydrochloric acid-induced cystitis in rats and that these effects are due, at least in part, to its anti-inflammatory effects on the bladder and inhibition of stretch-induced prostaglandin E_2_ release.

## Introduction

Lower urinary tract symptoms (LUTS), consisting of urinary retention, voiding, and post-voiding symptoms, are problematic because they can significantly impair the social activities of elderly individuals [1]. In particular, interstitial cystitis (IC), an intractable disease that causes frequent urination, urinary urgency, and bladder pain, can hinder workplace participation and lead to a decreased quality of life [2, 3]. Although the pathogenesis of IC has not been elucidated in detail, it has been suggested that inflammation and increased nociception play important roles [4–8]. In response to stretch stimulation, the bladders of patients with IC display an increase in the release of prostaglandin E_2_ (PGE_2_) [4, 5], which is involved in inflammation, fever, and pain, and has been shown to induce the urinary reflex via capsaicin-sensitive afferent nerves [6]. In addition, levels of the inflammatory cytokine interleukin (IL) 6 are elevated in patients with IC compared to healthy subjects and correlate positively with pain scores, indicating an association between inflammation and IC severity [7]. Hunners-type IC, which is associated with high levels of inflammation and high severity, can be successfully treated through intravesical injection with dimethyl sulfoxide (DMSO) [8] and the electrical cauterization of lesions [9], suggesting that bladder epithelium legions are involved in IC. Since PGE_2_ and IL6 are released from the bladder epithelium [10], it may be possible to improve the symptoms of cystitis like IC by suppressing bladder epithelium inflammation and the release of inflammatory molecules. Shiso (*Perilla frutescens var crispa f*. *purprea*) is a medicinal herb whose major polyphenol, rosmarinic acid (RA), has been reported to exert anti-inflammatory and antioxidant effects [11–14]. In addition, shiso extracts with a high RA content have been reported to be effective against mild LUTS in middle-aged and older adults [15]. Therefore, we hypothesized that RA may also be effective for treating cystitis including IC. In this study, we investigated the effects of RA on urinary function and the bladder in a rat model with hydrochloric acid (HCl)-induced cystitis, which has some features similar to those of IC including increased inflammation and bladder dysfunction [16, 17].

## Materials and methods

### Animals

Female Sprague-Dawley (SD) rats (11–12 weeks old, Japan SLC Inc., Shizuoka, Japan) were used in this study. All animals were provided with a standard diet (CE-2; CLEA Japan Inc., Tokyo, Japan) and water *ad libitum* and kept at 23 ± 2°C with a humidity of 55 ± 10% and a light-dark cycle of 12 h from 07:00–19:00. All animal experiments were approved by the Animal Care Committee of Kao Corporation (approval numbers: S19024-0000, S18007-0000) and were conducted according to its guidelines.

### Intravesical pressure measurement

To create models of cystitis, SD rats were randomly divided into three test groups (control group, HCl group, HCl + RA group; *n* = 7) with equal body weight [16, 17]. Under isoflurane anesthesia (Abbott Japan, Tokyo, Japan, #196756), the abdomen was incised to expose the bladder. A polyethylene catheter (PE50, Becton, Dickinson and Company, Franklin Lakes, NJ, USA, #427565) was implanted at the apex of the bladder and was used to inject 300 μL of 0.9% saline (Otsuka Pharmaceutical Co. Ltd., Tokyo, Japan, #3311401A7028; control group) or 0.1 N HCl (FUJIFILM Wako Pure Chemical Corp., Osaka, Japan, #081–01091; HCl and HCl + RA groups) into the bladder. After 10 min, the bladder was washed twice with 500 μL of saline solution, the catheter and bladder were sutured with silk thread, and the other end of the catheter was pulled out through the skin of the back of the neck and fixed outside the body. After surgery, each rat was kept individually in the environment described above. Rats were orally administered distilled water (control group, HCl group) or RA (50 mg/kg/day; Carbosynth Ltd., Berkshire, UK, #FR02310; HCl + RA group) under isoflurane inhalation anesthesia once a day immediately after surgery and for the following three days (a total of four times). The dose of RA was set by referring to previous studies in rats [18] and mice [19].

Three days after catheter implantation, intravesical pressure was measured in conscious rats. Briefly, the indwelling catheter in the bladder was connected to a transducer (Utah Medical Products Inc., Midvale, UT, USA, #6238) and a syringe pump (KD Scientific Inc., Holliston, MA, USA, #780100). Each rat was placed in a Ballman cage (W 46.5 mm (adjustable), D 160.0 mm, H 51.0 mm, Yamashitagiken Co. Ltd., Tokushima, Japan, #RTM2008) and urinary output was measured using an electromagnetic scale (GX-400, A&D Co. Ltd., Tokyo, Japan, #GX5400). Rats were not given food or water during intravesical pressure measurement. The bladder was infused with 0.9% saline using a syringe pump at a rate of 5 mL/h. After 20 min, the data for the 60 min following the first urination were analyzed. Micturition interval (sec), volume per voiding (mL), basal pressure (cmH_2_O), threshold pressure (cmH_2_O), and maximum pressure during voiding (cmH_2_O) were measured. Threshold pressure was calculated as the pressure in the bladder just before urinary contraction. Afterwards, the rats were euthanized under isoflurane anesthesia by cutting the abdominal vena cava and the bladder was harvested to measure its weight.

### Stretch stimulation using harvested bladders

SD rats were randomly divided into three experimental groups (control, HCl, HCl + RA; *n* = 7) with equal body weights and a catheter was inserted through the urethra. The cystitis model was prepared as described above and the test solution was orally administered once daily for three days (total of four times) until the day of euthanization. Approximately 1 h after the final oral administration, the rats were euthanized under isoflurane anesthesia by cutting the abdominal vena cava and were subjected to stretch stimulation as described previously [20]. After the right and left ureters of the bladder had been ligated using silk suturing thread, the bladder was harvested with approximately 5 mm of the urethra. The interior of the bladder and urethra were washed with Krebs solution (118 mM NaCl, 4.7 mM KCl, 2.5 mM CaCl_2_, 1.2 mM KH_2_PO_4_, 1.2 mM MgSO_4_, 25 mM NaHCO_3_, 11.1 mM D-glucose) and the bladder was weighed. Briefly, a catheter was inserted into the bladder through the urethra and the urethra was ligated. After catheter insertion, the bladder was acclimatized in a 10 mL tissue bath (World Precision Instruments, Sarasota, FL, USA, #47264) for 20 min under 95% O_2_, 5% CO_2_ conditions in Krebs solution at 37°C. After acclimation, 300 μL of Krebs solution was injected into the bladder through the catheter at a rate of 40 μL/s to measure baseline values. Ten minutes later, the injected solution was collected. After acclimation for 20 min, 600 μL or 900 μL of Krebs solution was injected into the bladder and the injected solution was collected after 10 min. After stretch stimulation, the bladder was cut into sections and were immersed in RNA later solution (Thermo Fisher Scientific, Bartlett, IL, USA, #AM7024) or OCT compound (Sakura Finetek Japan Co. Ltd., Tokyo, Japan, #4583). The collected Krebs solution and sections of the bladder were flash frozen in liquid nitrogen and stored at -80°C.

### Hematoxylin-eosin (H&E) staining

For staining, 8 μm-thick sections were prepared from frozen samples, fixed with 4% paraformaldehyde for 12 h at 4°C, and stained with H&E. To analyze the bladder epithelial layer and bladder wall length, three points were measured randomly per stained image using an all-in-one fluorescence microscope BZ-X710 (Keyence Corp., Osaka, Japan) and mean values were calculated under blind conditions.

### Myeloperoxidase (MPO) activity

MPO activity in the bladder was measured using an MPO activity assay kit (Abcam, Cambridge, UK, #ab105136) and normalized to the homogenate protein concentration. Absorbance was measured using a Viento XS multispectrophotometer (Sumitomo Pharma Co. Ltd., Osaka, Japan, #BTEONDN). Homogenate protein concentration was measured using a Pierce BCA Protein Assay Kit (Thermo Fisher Scientific, #23225).

### Cell culture

Normal human bladder epithelial cells (Kurabo Industries Ltd, Osaka, Japan, #KP-4309) were cultured in medium containing UroLife BM (Kurabo Industries, #LUB-LM0054) using a Urolife Comp kit (Kurabo Industries, #LUC-LL0071) at 37°C with 5% CO_2_. Cells were seeded in 12-well plates at a density of 5.0 × 10^4^ cells/well, pretreated with RA (Carbosynth) for 1 h at approximately 70% confluence, and then incubated with 10 ng/mL IL1β (FUJIFILM Wako Pure Chemical, #090–06121) for 4 h. After the supernatant had been collected, the cells were washed with phosphate buffered saline (PBS; FUJIFILM Wako Pure Chemical, #166–23555) and RNA was extracted using an RNeasy Mini Kit (Qiagen, Hilden, Germany, #74104).

### Real-time PCR (RT-PCR)

Bladder tissue sections (approximately 5 mm × 5 mm) were homogenized in ice-cold Tissue Protein Extraction Reagent (T-PER; Thermo Fisher Scientific, #78510) using a Physcotron NS-310EII (Microtec Co. Ltd., Chiba, Japan). Total RNA was extracted from homogenized bladder samples using an RNeasy Mini Kit (Qiagen). Total RNA (0.1 μg) extracted from bladder homogenates and bladder epithelial cells was reverse transcribed using a High Capacity RNA to cDNA kit (Thermo Fisher Scientific, #4387406). cDNA was subjected to RT-PCR using the following TaqMan probes (Thermo Fisher Scientific) with an Applied Biosystems 7500 Fast Real-Time PCR System (Thermo Fisher Scientific): *Actb* (Rn00667869_m1), *Cox2* (Rn01483828_m1), *Il6* (Rn01410330_m1), *ACTB* (Hs01060665_g1), *COX2* (Hs00153133_m1), *IL6* (Hs00174131_m1). Results were normalized to *Actb* or *ACTB*.

### Enzyme-linked immunosorbent assay (ELISA)

Bladder tissue sections (approximately 5 mm x 5 mm) were homogenized in ice-cold T-PER (Thermo Fisher Scientific) using a Physcotron NS-310EII (Microtec). The homogenate protein concentration was measured using a Pierce BCA Protein Assay Kit (Thermo Fisher Scientific). Absorbance was measured using a Viento XS multispectrophotometer (Sumitomo Pharma Co., Ltd.). Bladder IL6 protein levels were measured using a rat IL6 ELISA kit (Cusabio Technology LLC, Houston, TX, USA, #CSB-E04640r) and normalized to the homogenate protein concentration. The amount of PGE_2_ in the Krebs solution collected from the bladder stretch stimulation test was measured using a PGE_2_ Express EIA kit (Cayman Chemical, MI, Ann Arbor, USA, #500141) and normalized using bladder weight. The amount of IL6 in culture medium collected from bladder epithelial cells was measured using a human IL6 Quantikine ELISA kit (R&D systems, Minneapolis, MN, USA, #D6050).

### Statistical analysis

Data were presented as the mean ± the standard error of the mean (SEM). All data were analyzed using GraphPad Prism6 (GraphPad software Inc., San Diego, CA, USA). Mean values were compared between groups using Dunnett’s test. *P* values < 0.05 were considered significant.

## Results

### RA improves hydrochloric acid-induced shortening of micturition interval

**Fig 1:**
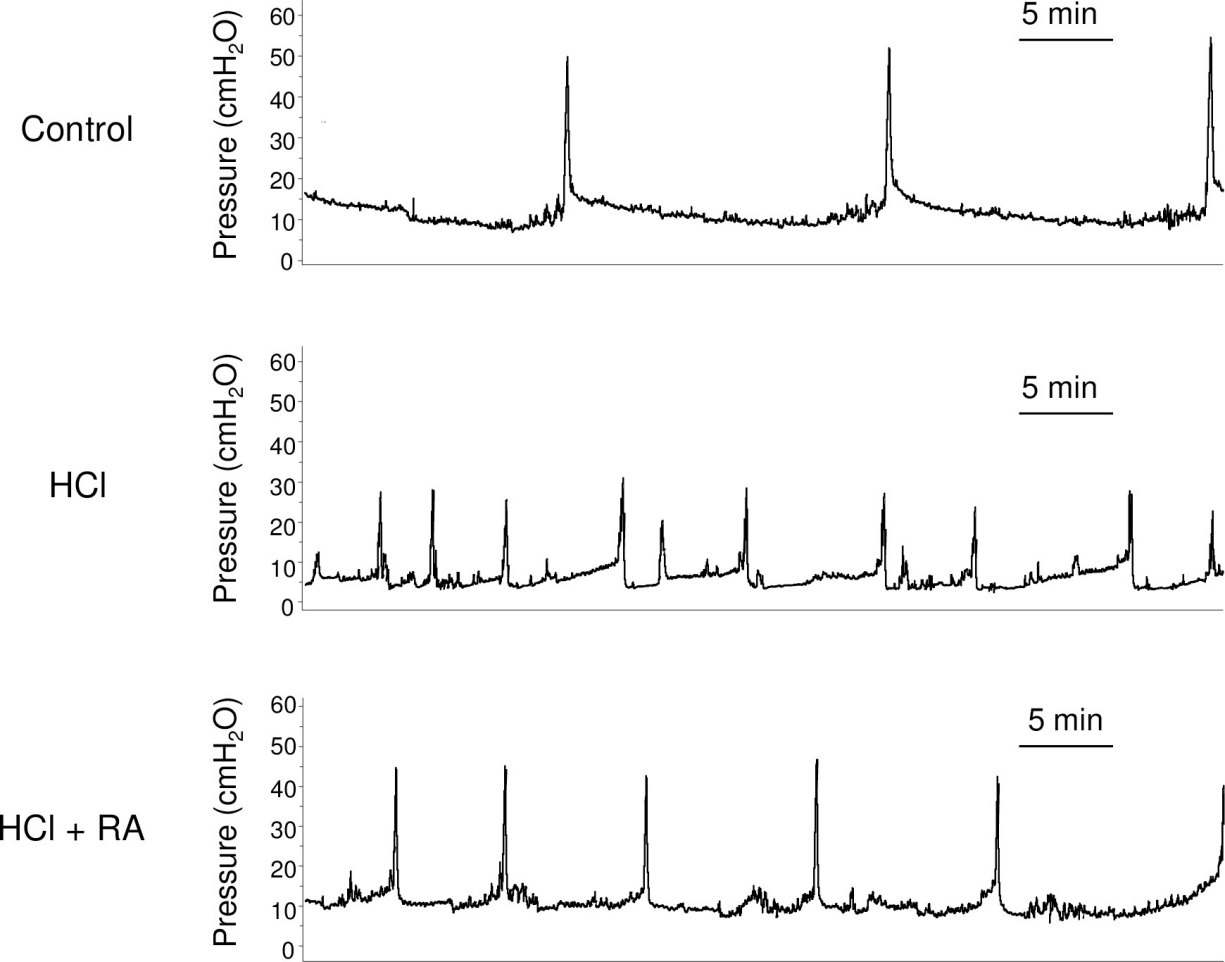
Representative cystometric charts of control, HCl-treated, and HCl + RA-treated rats. HCl, hydrochloric acid; RA, rosmarinic acid.

First, we examined changes in intravesical pressure in the rat model of cystitis (Fig 1). Micturition interval and volume per voiding were significantly decreased by 50% and 41%, respectively, in the HCl group compared to the control group (Table 1; *p* < 0.001 and *p* = 0.004, respectively). Conversely, the micturition interval was significantly increased by 49% in the HCl + RA group compared to the HCl group (Table 1; *p* = 0.017). No significant changes in micturition volume, basal pressure, threshold pressure, or maximal pressure during voiding were observed between the different experimental groups.

**Table 1:** Cystometric analysis in control, HCl-treated, and HCl + RA-treated rats.

	Control	HCl	HCl + RA
**Micturition interval (sec)**	908.9 ± 42.9***	456.6 ± 25.6	678.3 ± 77.9*
**Micturition volume (mL)**	1.14 ± 0.09**	0.67 ± 0.07	0.92 ± 0.11
**Basal pressure (cmH**_**2**_ **O)**	9.26 ± 2.31	9.35 ± 2.18	12.30 ± 2.28
**Threshold pressure (cmH**_**2**_ **O)**	18.70 ± 1.70	18.01 ± 2.46	23.30 ± 3.14
**Maximal pressure (cmH**_**2**_ **O)**	54.94 ± 4.85	48.58 ± 5.47	55.78 ± 2.85

Data represent the mean ± SEM (*n* = 7)

*p < 0.05

**p < 0.01

***p < 0.001 vs. the HCl group, Dunnett’s test. Abbreviations: HCl, hydrochloric acid; RA, rosmarinic acid.

### RA inhibits PGE_2_ release in response to stretch stimulation

Next, we measured the effect of RA on PGE_2_ release from the luminal side after the bladder had been stretched by intravesical injection with Krebs solution. After stimulation with 600 μL of Krebs solution, PGE_2_ release was approximately 10-fold higher in the HCI group than in the control group (Table 2; *p* = 0.026) but was suppressed to control levels in the HCl + RA group (Table 2; *p* = 0.021). Although intravesical injection with 900 μL Krebs solution resulted in similar trends in PGE_2_ release, the differences between the three groups were not significant (Table 2).

**Table 2:** Stretch-induced prostaglandin E_2_ release in control, HCl-treated, and HCl + RA-treated rats.

Infusion volume	Control	HCl	HCl + RA
**600 μL**	179.7 ± 138.6*	1822.0 ± 721.3	121.9 ± 91.1*
**900 μL**	663.0 ± 290.4	2331.0 ± 1267.5	505.1 ± 250.2

Data represent the mean ± SEM (*n* = 7, ng/g tissue)

*p < 0.05 vs. the HCl group, Dunnett’s test. Abbreviations: HCl, hydrochloric acid; RA, rosmarinic acid.

### RA improves inflammatory conditions in the bladder

**Fig 2:**
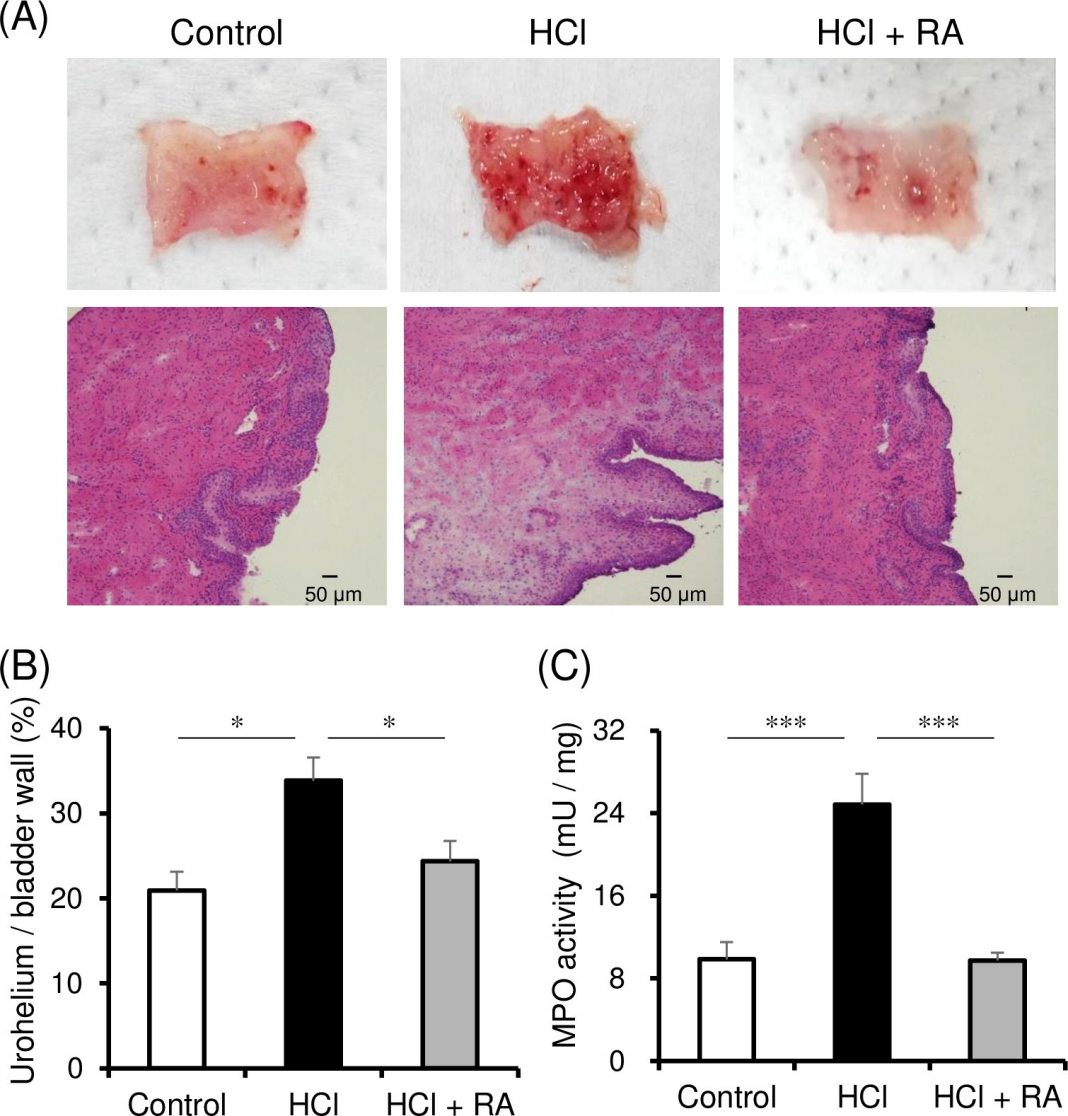
Histopathological and biochemical evaluation of rat bladder. Representative hematoxylin-eosin stained sections (A), mean urothelial thickness (B, Control; n = 5, HCl; n = 6, HCl + RA; n = 7), and mean MPO activity (C, n = 7, respectively) in control, HCl-treated, and HCl + RA-treated bladders. Mean ± SEM; significant difference from the HCl group; Dunnett’s test, *P < 0.05, ***P < 0.001. HCl, hydrochloric acid; MPO, myeloperoxidase; RA, rosmarinic acid.

The rats subjected to intravesical pressure measurement and the stretch stimulation test displayed no difference in body weight among groups (*n* = 14/group; control: 217.0 ± 3.8 g, HCl: 220.9 ± 4.5 g, and HCI + RA: 220.3 ± 4.5 g). However, bladder weight increased to 204.4 ± 10.1 mg in the HCl group (*p* < 0.001) and 169.4 ± 6.5 mg in the HCl + RA group compared to the control group (138.1 ± 4.3 mg). In addition, histological analysis revealed redness and thickening of the epithelial layer in HCl-treated rats compared to control rats. After HCl treatment, the percentage of the epithelial layer to the bladder wall increased from 21% to 34% (Fig 2A and 2B; *p* = 0.003); however, epithelial layer redness and thickening were reduced in the HCl + RA group compared to the HCl group and the percentage of the epithelial layer to the bladder wall decreased from 34% to 24% (Fig 2A and 2B; *p* = 0.026). Furthermore, we found that MPO activity [21], which indicates the presence of neutrophils [22], was approximately 2.5-fold higher in the HCl group than in the control group (Fig 2C; *p* < 0.001) but was decreased to control levels in the HCl + RA group (Fig 2C; *p* < 0.001).

### RA inhibits the induction of inflammation-related molecules in the bladder and in bladder epithelial cells

**Fig 3:**
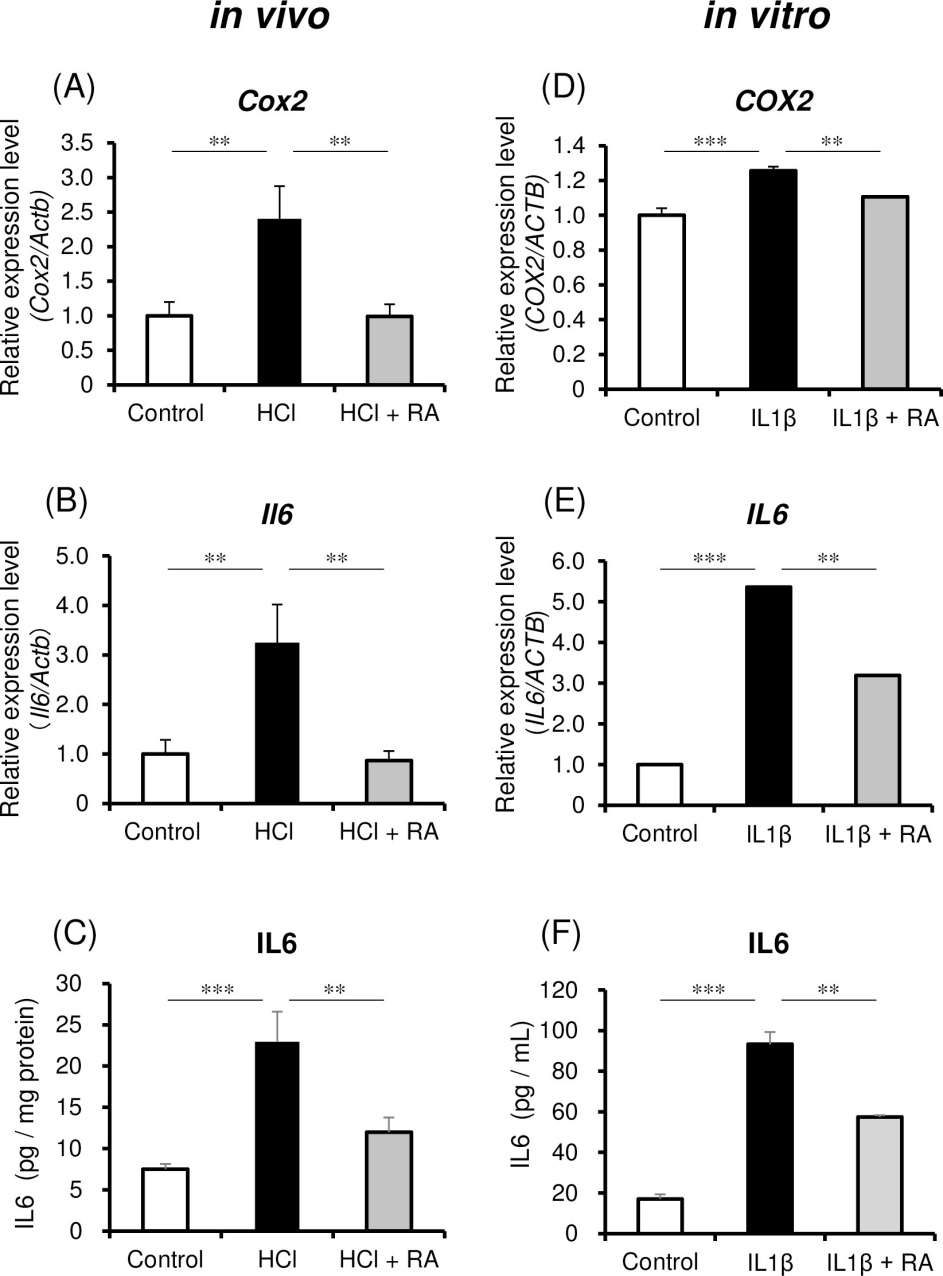
Effect of RA on the expression of inflammatory molecules. Relative mRNA expression levels of *Cox2* (A) and *Il6* (B), and protein levels of IL6 (C) in control, HCl-treated, and HCl + RA-treated bladders. Data represent the mean ± SEM (*n* = 7). Relative mRNA expression levels of *COX2* (D) and *IL6* (E) in human bladder epithelial cells and amount of IL6 released into the supernatant (F) after treatment. Data represent the mean ± SEM (*n* = 3). mRNA expression was normalized to *Actb* or *ACTB* relative to the control group; **p < 0.01, ***p < 0.001 vs. the HCl or IL1β groups, Dunnett’s test. HCl, hydrochloric acid; IL1β, interleukin 1β; RA, rosmarinic acid.

Finally, we examined the effect of RA on the expression of inflammation-related molecules in models of cystitis *in vivo* and *in vitro*. In the bladders of rats treated with HCl, *Cox2* and *Il6* expression levels were increased (Fig 3A and 3B; *p* = 0.010 and *p* = 0.009, respectively); however, this increase was significantly suppressed by RA administration (Fig 3A and 3B; *p* = 0.009 and *p* = 0.006, respectively). In addition, IL6 levels were higher in bladders from the HCl group than from the control group (Fig 3C; *p* < 0.001), but were suppressed in the HCl + RA group (Fig 3C; *p* = 0.008). In human bladder epithelial cells treated with IL1β, which is abundant in the bladder of rat model of cystitis and is known to induce IL6 and COX2 [12, 13], *COX2* and *IL6* expression were significantly increased (Fig 3D and 3E; each *p* < 0.001); however, pretreatment with RA for 1 h significantly suppressed the IL1β-induced increase in *COX2* and *IL6* expression (Fig 3D and 3E; *p* = 0.005 and *p* = 0.001, respectively). Similarly, the addition of IL1β increased the release of IL6 from bladder epithelial cells into the culture medium and this increase was suppressed by pretreatment with RA (Fig 3F; *p* < 0.001 and *p* = 0.001, respectively).

## Discussion

RA has been reported to exert anti-inflammatory and anti-oxidant effects; however, its effects against cystitis with bladder inflammatory changes and dysfunction remain unclear. In this study, we showed that RA improved HCl-induced cystitis in rats, possibly by acting against the bladder epithelium to suppress the production of inflammatory mediators and inhibited PGE_2_ release in response to stretch stimulation. Furthermore, we found that oral administration with RA improved HCl-induced shortening of micturition interval without affecting the volume per voiding, basal pressure, threshold pressure, or maximal pressure during voiding, suggesting that RA improves HCl-induced cystitis by acting on the bladder epithelium rather than bladder smooth muscle.

Although the detailed pathogenesis of cystitis including IC remains unclear, it is thought to involve inflammation [4–8]. Rat models of cystitis typically display thickening of the bladder epithelial layer [16], increased MPO activity [21], and the induction of proinflammatory cytokines [23]. In this study, we found that RA administration suppressed a series of inflammatory changes induced by HCl treatment, suggesting that RA may improve the shortening of micturition interval by suppressing inflammation and associated epithelial thickening in the bladder.

During the acute phase of inflammation, neutrophils infiltrate tissues and produce various inflammatory mediators, including IL1β and IL6 [24]. In addition, stimulating bladder epithelial cells with IL1β induces the expression and release of IL6. Thus, IL6 is produced in the bladder epithelial and stromal layers [7] and is thought to play a role in cystitis development [25]. Indeed, it has been reported that urinary IL6 levels are higher in patients with IC than in healthy individuals [7]. In this study, we found that RA administration suppressed increased IL6 production and MPO activity, which is associated with neutrophil infiltration, in bladder tissue. Thus, RA may decrease IL6 production to improve cystitis symptoms including shortening of micturition interval. Since IL6 receptors are expressed on sensory nerves and induce hyperalgesia, suggesting that IL6 is involved in afferent neurotransmission [26], IL6 in the bladder may act against IL6 receptors on sensory nerves to transmit abnormal urges to urinate which are associated with cystitis. Consequently, RA may suppress the non-mechanosensitive transmission of signals involved in urinary urgency.

The bladder epithelium is thought to be the lesion responsible for cystitis since intravesical DMSO injection [8] and electrical lesion cautery [9] have shown efficacy in Hunners-type IC. After oral ingestion, RA is present in the blood and urine of humans and rats [18, 27], suggesting that orally administered RA can act on the bladder epithelium via both the blood and urine. When we examined the direct effects of RA using human bladder epithelial cells, we found that *IL6* expression and IL6 production by IL1β were significantly inhibited by RA. Together, these results suggest that RA can exert a series of effects against bladder epithelium, including inhibiting IL6 production. Previous studies in models of inflammatory diseases, including colitis, pancreatitis, and asthma, have reported that RA can suppress pro-inflammatory cytokine production and the activation of NF-κB, an upstream transcription factor [28]. Here, we found that RA suppressed *IL6* and *COX2* expression, both of which have NF-κB consensus sequences in their promoter regions [29], in bladders and bladder epithelial cells, suggesting that RA may suppress inflammatory mediator expression and subsequent bladder inflammation at the transcriptional level via inhibition of NF-κB activation.

PGE_2_, which is synthesized from arachidonic acid by phospholipase A2 and COX, has vasodilatory effects and plays an important role in the inflammatory response [4]. Urinary PGE_2_ levels are increased in patients with IC [5] and intravesical PGE_2_ administration decrease bladder capacity and increase the frequency of urination [30]. In addition, PGE_2_ is released from the bladder in response to stretch stimulation [20] and is involved in the transmission of signals related to the urge to urinate [6]. These findings suggest that abnormal PGE_2_ production may be a cause of bladder dysfunction. In the rat model, the amount of PGE_2_ released from the harvested bladder during stretch stimulation was significantly increased by HCl treatment, whereas RA suppressed PGE_2_ release. Furthermore, RA suppressed HCl-induced *Cox2* expression in the bladder and IL1β-induced *COX2* expression in cultured bladder epithelial cells, suggesting that RA inhibits PGE_2_ synthesis and release, and subsequent mechanosensitive urinary transmission. It has been reported that parecoxib, a COX2 inhibitor, can suppress COX expression and improve cystitis in rats [29]. This also supports the possibility that RA improves cystitis by suppressing COX expression and inhibiting stretch-induced PGE_2_ release.

In this study, RA was administered three days after HCl treatment; therefore, our findings reflect the effects of RA during the acute phase of cystitis. Consequently, future studies should clarify the efficacy of RA on cystitis in animal models with chronic as well as acute inflammation and the detailed mechanisms involving NF-κB inhibition.

Since lower urinary tract obstructions such as benign prostatic hyperplasia are often associated with urinary retention in older adults, it is necessary to improve urinary retention symptoms without reducing the contractility of bladder smooth muscle [31]. Considering the point of action of RA, Shiso extract, which contains high levels of RA and has been shown to improve urinary storage function [15], may be effective for improving LUTS with lower urinary tract obstruction in middle-aged and older adults.

## Conclusion

Taken together, the findings of this study demonstrate that RA ameliorates HCl-induced cystitis in rats by exerting anti-inflammatory effects in the bladder. Thus, RA, which can be consumed on a daily basis, could be an effective way to improve lower urinary tract symptoms, including cystitis.

## Supporting information

S1 Table Mean urothelial thickness of rat bladder. Data represent the mean ± SEM (Control; n = 5, HCl; n = 6, HCl + RA; n = 7); HCl, hydrochloric acid; RA, rosmarinic acid.
(DOCX)

S2 Table Mean MPO activity in rat bladder. Data represent the mean ± SEM (n = 7); HCl, hydrochloric acid; MPO, myeloperoxidase; RA, rosmarinic acid.
(DOCX)

S3 Table Relative mRNA expression of *Cox2* and *Il6* in rat bladder. Data represent the mean ± SEM (n = 3); The amount of each mRNA was normalized to *Actb* mRNA levels and expressed relative to the control group. HCl, hydrochloric acid; RA, rosmarinic acid.
(DOCX)

S4 Table Mean content of IL6 in rat bladder. Data represent the mean ± SEM (n = 7); HCl, hydrochloric acid; RA, rosmarinic acid.
(DOCX)

S5 Table Relative mRNA expression of *COX2* and *IL6* in human bladder epithelial cells. Data represent the mean ± SEM (n = 3); The amount of each mRNA was normalized to *ACTB* mRNA levels and expressed relative to the control group. IL1β, interleukin 1β; RA, rosmarinic acid.
(DOCX)

S6 Table Mean value of IL6 released into supernatant from human bladder epithelial cells. Data represent the mean ± SEM (n = 7); IL1β, interleukin 1β; RA, rosmarinic acid.
(DOCX)

## References

[1] Homma Y, Yamaguchi O, Hayashi K, Neurogenic Bladder Society Committee. Epidemiologic survey of lower urinary tract symptoms in Japan. Urology. 2006;68: 560–564. doi: 10.1016/j.urology.2006.03.035 16979726

[2] Nickel JC, Tripp DA, Pontari M, Moldwin R, Mayer R, Carr LK, et al. Psychosocial phenotyping in women with interstitial cystitis/painful bladder syndrome: A case control study. J Urol. 2010;183: 167–172. doi: 10.1016/j.juro.2009.08.133 19913812

[3] Beckett MK, Elliott MN, Clemens JQ, Ewing B, Berry SH. Consequences of interstitial cystitis/bladder pain symptoms on women’s work participation and income: Results from a national household sample. J Urol. 2014;191: 83–88. doi: 10.1016/j.juro.2013.07.018 23872030 PMC4085039

[4] Narumiya S, Sugimoto Y, Ushikubi F. Prostanoid receptors: structures, properties, and functions. Physiol Rev. 1999;79: 1193–1226. doi: 10.1152/physrev.1999.79.4.1193 10508233

[5] Wada N, Ameda K, Furuno T, Okada H, Date I, Kakizaki H. Evaluation of prostaglandin E_2_ and E-series prostaglandin receptor in patients with interstitial cystitis. J Urol. 2015;193: 1987–1993. doi: 10.1016/j.juro.2015.01.010 25595860

[6] Maggi CA, Giuliani S, Conte B, Furio M, Santicioli P, Meli P, et al. Prostanoids modulate reflex micturition by acting through capsaicin-sensitive afferents. Eur J Pharmacol. 1988;145: 105–112. doi: 10.1016/0014-2999(88)90221-x 3162418

[7] Lotz M, Villiger P, Hugli T, Koziol J, Zuraw BL. Interleukin-6 and interstitial cystitis. J Urol. 1994;152: 869–873. doi: 10.1016/s0022-5347(17)32594-6 8051739

[8] Peeker R, Haghsheno MA, Holmäng S, Fall M. Intravesical bacillus Calmette-Guerin and dimethyl sulfoxide for treatment of classic and nonulcer interstitial cystitis: A prospective, randomized double-blind study. J Urol. 2000;164: 1912–1915; discussion 1915–1916. doi: 10.1016/s0022-5347(05)66916-9 11061879

[9] Kerr WS Jr. Interstitial cystitis: Treatment by transurethral resection. J Urol. 1971;105: 664–666. doi: 10.1016/s0022-5347(17)61602-1 4397018

[10] McDermott C, Chess-Williams R, Mills KA, Kang SH, Farr SE, Grant GD, et al. Alterations in acetylcholine, PGE2 and IL6 release from urothelial cells following treatment with pyocyanin and lipopolysaccharide. Toxicol In Vitro. 2013;27: 1693–1698. doi: 10.1016/j.tiv.2013.04.015 23665401

[11] Takano H, Osakabe N, Sanbongi C, Yanagisawa R, Inoue K, Yasuda A, et al. Extract of Perilla frutescens enriched for rosmarinic acid, a polyphenolic phytochemical, inhibits seasonal allergic rhinoconjunctivitis in humans. Exp Biol Med (Maywood). 2004;229: 247–254. doi: 10.1177/153537020422900305 14988517

[12] Hu ZN, Huang LJ, Chen WP. The inhibitory effects of rosmarinic acid on catabolism induced by IL-1β in rat chondrocyte. Acta Biochim Pol. 2018;65: 535–538. doi: 10.18388/abp.2018_2607 30534634

[13] Chen WP, Jin GJ, Xiong Y, Hu PF, Bao JP, Wu LD. Rosmarinic acid down-regulates NO and PGE_2_ expression via MAPK pathway in rat chondrocytes. J Cell Mol Med. 2018;22: 346–353. doi: 10.1111/jcmm.13322 28945000 PMC5742733

[14] Park JB. Identification and quantification of a major anti-oxidant and anti-inflammatory phenolic compound found in basil, lemon thyme, mint, oregano, rosemary, sage, and thyme. Int J Food Sci Nutr. 2011;62: 577–584. doi: 10.3109/09637486.2011.562882 21506887

[15] Kitamura N, Yamamoto N, Yokoyama O, Murase T. Perilla extract improves lower urinary tract symptoms and sleep in Japanese adults–A randomized placebo-controlled crossover study. Jpn Pharmacol Ther. 2020;48: 2145–2152.

[16] Kitta T, Tanaka H, Mitsui T, Moriya K, Nonomura K. Type 4 phosphodiesterase inhibitor suppresses experimental bladder inflammation. BJU Int. 2008;102: 1472–1476. doi: 10.1111/j.1464-410X.2008.07662.x 18410434

[17] Rivas DA, Chancellor MB, Shupp-Byrne S, Shenot PJ, McHugh K, McCue P. Molecular marker for development of interstitial cystitis in rat model: isoactin gene expression. J Urol. 1997;157: 1937–1940. doi: 10.1016/s0022-5347(01)64905-x 9112567

[18] Baba S, Osakabe N, Natsume M, Terao J. Orally administered rosmarinic acid is present as the conjugated and/or methylated forms in plasma, and is degraded and metabolized to conjugated forms of caffeic acid, ferulic acid and m-coumaric acid. Life Sci. 2004;75: 165–178. doi: 10.1016/j.lfs.2003.11.028 15120569

[19] Domitrovic R, Skoda M, Marchesi VV, Cvijanovic O, Pugel EP, Stefan MB. Rosmarinic acid ameliorates acute liver damage and fibrogenesis in carbon tetrachloride-intoxicated mice. Food Chem Toxicol. 2013;51: 370–378. doi: 10.1016/j.fct.2012.10.021 23116643

[20] Tanaka I, Nagase K, Tanase K, Aoki Y, Akino H, Yokoyama O. Modulation of stretch evoked adenosine triphosphate release from bladder epithelium by prostaglandin E_2_. J Urol. 2011;185: 341–346. doi: 10.1016/j.juro.2010.09.042 21075387

[21] Sahiner IF, Soylu H, Ates E, Acar N, Ustunel I, Danisman A. Impact of intravesical hyaluronic acid treatment on bladder inflammation in interstitial cystitis rat model. Int Braz J Urol. 2018;44: 1014–1022. doi: 10.1590/S1677-5538.IBJU.2017.0713 30044599 PMC6237519

[22] Bradley PP, Priebat DA, Christensen RD, Rothstein G. Measurement of cutaneous inflammation: estimation of neutrophil content with an enzyme marker. J Invest Dermatol. 1982;78: 206–209. doi: 10.1111/1523-1747.ep12506462 6276474

[23] Funahashi Y, Yoshida M, Yamamoto T, Majima T, Takai S, Gotoh M. Intravesical application of rebamipide promotes urothelial healing in a rat cystitis model. J Urol. 2014;192: 1864–1870. doi: 10.1016/j.juro.2014.06.081 24992331

[24] Dornelas-Filho AF, Pereira VBM, Wong DVT, Nobre LMS, Melo AT, Silva CMS, et al. Neutrophils contribute to the pathogenesis of hemorrhagic cystitis induced by ifosfamide. Int Immunopharmacol. 2018;62: 96–108. doi: 10.1016/j.intimp.2018.06.031 29990699

[25] Zheng Z, Zhang J, Zhang C, Li W, Ma K, Huang H et al. The study on the function and cell source of interleukin-6 in interstitial cystitis/bladder painful syndrome rat model. Immun Inflamm Dis. 2021;9: 1520–1528. doi: 10.1002/iid3.505 34407316 PMC8589393

[26] Cunha FQ, Poole S, Lorenzetti BB, Ferreira SH. The pivotal role of tumour necrosis factor alpha in the development of inflammatory hyperalgesia. Br J Pharmacol. 1992;107: 660–664. doi: 10.1111/j.1476-5381.1992.tb14503.x 1472964 PMC1907751

[27] Baba S, Osakabe N, Natsume M, Yasuda A, Muto Y, Hiyoshi K, et al. Absorption, metabolism, degradation and urinary excretion of rosmarinic acid after intake of *Perilla frutescens* extract in humans. Eur J Nutr. 2005;44: 1–9. doi: 10.1007/s00394-004-0482-2 15309457

[28] Luo C, Zou L, Sun H, Peng J, Gao C, Bao L, et al. A review of the anti-inflammatory effects of rosmarinic acid on inflammatory diseases. Front Pharmacol. 2020;11: 153. doi: 10.3389/fphar.2020.00153 32184728 PMC7059186

[29] Juan YS, Lee YL, Long CY, Wong JH, Jang MY, Lu JH, et al. Translocation of NF-κB and expression of cyclooxygenase-2 are enhanced by ketamine-induced ulcerative cystitis in rat bladder. Am J Pathol. 2015;185: 2269–2285. doi: 10.1016/j.ajpath.2015.04.020 26073037

[30] Ishizuka O, Mattiasson A, Andersson KE. Prostaglandin E2-induced bladder hyperactivity in normal, conscious rats: involvement of tachykinins? J Urol. 1995;153: 2034–2038. doi: 10.1016/s0022-5347(01)67397-x 7752389

[31] Lee JY, Kim HW, Lee SJ, Koh JS, Suh HJ, Chancellor MB. Comparison of doxazosin with or without tolterodine in men with symptomatic bladder outlet obstruction and an overactive bladder. BJU Int. 2004;94: 817–820. doi: 10.1111/j.1464-410X.2004.05039.x 15476515

