# Supplementary material for: Rosmarinic acid ameliorates HCl-induced cystitis in rats
Source: PLoS One. 2023 Jul 18;18(7):e0288813. doi: 10.1371/journal.pone.0288813 (PMC10353813; doi:10.1371/journal.pone.0288813)
Supplement: S1 Table — Data represent the mean ± SEM (Control; n = 5, HCl; n = 6, HCl + RA; n = 7); HCl, hydrochloric acid; RA, rosmarinic acid. (DOCX) [file pone.0288813.s001.docx]

**S1 Table.** **Mean urothelial thickness of rat bladder.**

|  | **Control** | **HCl** | **HCl + RA** |
| --- | --- | --- | --- |
| **Urohelium /**  **bladder wall (%)** | 20.9 ± 3.0 | 33.9 ± 3.2 | 24.4 ± 2.4 |

Data represent the mean ± SEM (Control; n = 5, HCl; n = 6, HCl + RA; n = 7); HCl, hydrochloric acid; RA, rosmarinic acid.
