# Supplementary material for: Rosmarinic acid ameliorates HCl-induced cystitis in rats
Source: PLoS One. 2023 Jul 18;18(7):e0288813. doi: 10.1371/journal.pone.0288813 (PMC10353813; doi:10.1371/journal.pone.0288813)
Supplement: S2 Table — Data represent the mean ± SEM (n = 7); HCl, hydrochloric acid; MPO, myeloperoxidase; RA, rosmarinic acid. (DOCX) [file pone.0288813.s002.docx]

**S2 Table.** **Mean MPO activity in rat bladder.**

|  | **Control** | **HCl** | **HCl + RA** |
| --- | --- | --- | --- |
| **MPO activity**  **(mU / mg)** | 9.8 ± 1.7 | 24.8 ± 3.0 | 9.7 ± 0.8 |

Data represent the mean ± SEM (n = 7); HCl, hydrochloric acid; MPO, myeloperoxidase; RA, rosmarinic acid.
