# Supplementary material for: Rosmarinic acid ameliorates HCl-induced cystitis in rats
Source: PLoS One. 2023 Jul 18;18(7):e0288813. doi: 10.1371/journal.pone.0288813 (PMC10353813; doi:10.1371/journal.pone.0288813)
Supplement: S3 Table — Data represent the mean ± SEM (n = 3); The amount of each mRNA was normalized to Actb mRNA levels and expressed relative to the control group. HCl, hydrochloric acid; RA, rosmarinic acid. (DOCX) [file pone.0288813.s003.docx]

**S3 Table. Relative mRNA expression of *Cox2* and *Il6*** **in rat bladder.**

|  | **Control** | **HCl** | **HCl + RA** |
| --- | --- | --- | --- |
| ***Cox2*** | 1.00 ± 0.20 | 2.40 ± 0.47 | 0.99 ± 0.17 |
| ***Il6*** | 1.00 ± 0.29 | 3.25 ± 0.77 | 0.87 ± 0.20 |

Data represent the mean ± SEM (n = 3); The amount of each mRNA was normalized to *Actb* mRNA levels and expressed relative to the control group. HCl, hydrochloric acid; RA, rosmarinic acid.
