# Supplementary material for: Rosmarinic acid ameliorates HCl-induced cystitis in rats
Source: PLoS One. 2023 Jul 18;18(7):e0288813. doi: 10.1371/journal.pone.0288813 (PMC10353813; doi:10.1371/journal.pone.0288813)
Supplement: S4 Table — Data represent the mean ± SEM (n = 7); HCl, hydrochloric acid; RA, rosmarinic acid. (DOCX) [file pone.0288813.s004.docx]

**S4 Table.** **Mean content of IL6 in rat bladder.**

|  | **Control** | **HCl** | **HCl + RA** |
| --- | --- | --- | --- |
| **IL6**  **(pg / mg protein)** | 7.5 ± 0.6 | 22.9 ± 3.7 | 12.0 ± 1.8 |

Data represent the mean ± SEM (n = 7); HCl, hydrochloric acid; RA, rosmarinic acid.
