# Supplementary material for: Rosmarinic acid ameliorates HCl-induced cystitis in rats
Source: PLoS One. 2023 Jul 18;18(7):e0288813. doi: 10.1371/journal.pone.0288813 (PMC10353813; doi:10.1371/journal.pone.0288813)
Supplement: S5 Table — Data represent the mean ± SEM (n = 3); The amount of each mRNA was normalized to ACTB mRNA levels and expressed relative to the control group. IL1β, interleukin 1β; RA, rosmarinic acid. (DOCX) [file pone.0288813.s005.docx]

**S5 Table. Relative mRNA expression of *COX2* and *IL6* in human bladder epithelial cells.**

|  | **Control** | **IL1β** | **IL1β + RA** |
| --- | --- | --- | --- |
| ***COX2*** | 1.00 ± 0.04 | 1.26 ± 0.02 | 1.11 ± 0.01 |
| ***IL6*** | 1.00 ± 0.01 | 5.36 ± 0.16 | 3.19 ± 0.02 |

Data represent the mean ± SEM (n = 3); The amount of each mRNA was normalized to *ACTB* mRNA levels and expressed relative to the control group. IL1β, interleukin 1β; RA, rosmarinic acid.
