# Supplementary material for: Rosmarinic acid ameliorates HCl-induced cystitis in rats
Source: PLoS One. 2023 Jul 18;18(7):e0288813. doi: 10.1371/journal.pone.0288813 (PMC10353813; doi:10.1371/journal.pone.0288813)
Supplement: S6 Table — Data represent the mean ± SEM (n = 7); IL1β, interleukin 1β; RA, rosmarinic acid. (DOCX) [file pone.0288813.s006.docx]

**S6 Table.** **Mean value of IL6 released into supernatant from human bladder epithelial cells.**

|  | **Control** | **IL1β** | **IL1β + RA** |
| --- | --- | --- | --- |
| **IL6**  **(pg / mL)** | 17.0 ± 2.4 | 93.3 ± 6.1 | 57.5 ± 0.9 |

Data represent the mean ± SEM (n = 7); IL1β, interleukin 1β; RA, rosmarinic acid.
